# A Universal Strategy for Stretchable Polymer Nonvolatile Memory via Tailoring Nanostructured Surfaces

**DOI:** 10.1038/s41598-019-46884-4

**Published:** 2019-07-17

**Authors:** Chaoyi Ban, Xiangjing Wang, Zhe Zhou, Huiwu Mao, Shuai Cheng, Zepu Zhang, Zhengdong Liu, Hai Li, Juqing Liu, Wei Huang

**Affiliations:** 1 Key Laboratory of Flexible Electronics (KLOFE) & Institute of Advanced Materials (IAM), Nanjing Tech University (NanjingTech), 30 South Puzhu Road, Nanjing, 211816 China; 2 Shaanxi Institute of Flexible Electronics (SIFE), Northwestern Polytechnical University (NPU), 127 West Youyi Road, Xi’an, 710072 China; 3 Key Laboratory for Organic Electronics and Information Displays & Institute of Advanced Materials (IAM), SICAM, Nanjing University of Posts & Telecommunications, 9 Wenyuan Road, Nanjing, 210023 China

**Keywords:** Optical properties and devices, Electronic devices

## Abstract

Building stretchable memory is an effective strategy for developing next-generation memory technologies toward stretchable and wearable electronics. Here we demonstrate a universal strategy for the fabrication of high performance stretchable polymer memory via tailoring surface morphology, in which common conjugated polymers and sharp reduced graphene oxide (r-rGO) films are used as active memristive layers and conductive electrodes, respectively. The fabricated devices feature write-once-read-many-times (WORM) memory, with a low switching voltage of 1.1 V, high ON/OFF current ratio of 10^4^, and an ideal long retention time over 12000 s. Sharp surface-induced resistive switching behavior has been proposed to explore the electrical transition. Moreover, the polymer memory show reliable electrical bistable properties with a stretchability up to 30%, demonstrating their great potential candidates as high performance stretchable memory in soft electronics.

## Introduction

Polymer memory, a promising emerging memory technology, have captured great attention owing to their fast operating speed, low power consumption, simple structure and high integrated density^1^. Specially, the intrinsically flexible properties of polymers endow polymer memory with high flexibility, enabling them as basic building blocks in flexible electronics and skin electronics for data storage^2–4^, logic, neuromorphic computing^5^ and artificial synapses^6,7^. In the past decade, flexible memory materials mainly include polymer composites and pure polymers comprising complex structures, such as donor-acceptor and conformational-change systems^7^. Flexible electrodes normally consist of graphene or reduced graphene oxide (rGO)^7,8^, conductive polymer^9^, silver nanowire and several metal conductors^7,10^. Despite extensive effort devoted to flexible polymer memory, the stretchability of memory devices has not been well solved, limiting their uses for growing demands in stretchable electronics, wearable electronics and smart electronics.

In the pursuit of memory devices with high stretchability, several strategies have been exploited to retain the electrical hysteresis behaviors of memory device under tensile deformation. The prevailing approach is to design device array with stretchable interconnectors between the individual rigid devices^11–20^. For example, Kim *et al*. reported a stretchable carbon nanotube charge-trap floating-gate memory arrays by integrating rigid transistor memory device and logic gates with serpentine interconnections for enhanced deformability of the integrated system^20^. Another strategy is to shape rigid materials with mechanically deformable structures on elastic substrates to achieve stretchable floating-gate memories, such as buckled-, spring-, and meshed-structures^21–24^. Recently, Chen *et al*. demonstrated a stretchable organic nonvolatile memory diode with buckled structure by transferring flexible compounds on pre-strained elastomer, with mixed semiconducting/insulating polymers as active layer^25^. Such research facilitates the development of data storage devices toward stretchable electronic applications. However, these strategies face the challenges, for example, complex fabrication process owing to their complicated structures or multicomponent of memristive materials, impeding their universal and low cost manufacturing as well as high density integration. Therefore, developing a general strategy to fabricate stretchable polymer memory devices with diverse common materials and simple diode structure is highly desirable.

Here we report a facile and universal strategy for high-throughput fabrication of high performance stretchable polymer memory device by direct depositing polymer diode onto a pre-strained poly(dimethylsiloxane) (PDMS) elastic substrate. By utilizing pure conjugated polymer Poly(9-vinylcarbazole (PVK) active layer sandwiched between alumina (Al) top electrode and rough reduced graphene oxide (r-rGO) bottom electrode, the as-fabricated polymer device exhibits typical electrical bistable behavior and nonvolatile memory effect, with the merits of high ON/OFF ratio, low switching voltage, outstanding retention ability and excellent reproducibility. Moreover, the memory device possesses reliable ability to operate under uniaxial tensile strain. Meanwhile, we have also discussed the carrier transport process and memory mechanism. Importantly, this method is general and can be used to prepare other stretchable polymer memory devices comprising pure conjugated polymers, including poly[2-methoxy-5-(2-ethylhexyloxy) phenylenevinylene-1,4-diyl] (MEH:PPV) and poly(9, 9-di-n-octylfluorenyl-2,7-diyl) (PFO).

## Results

**Figure 1:**
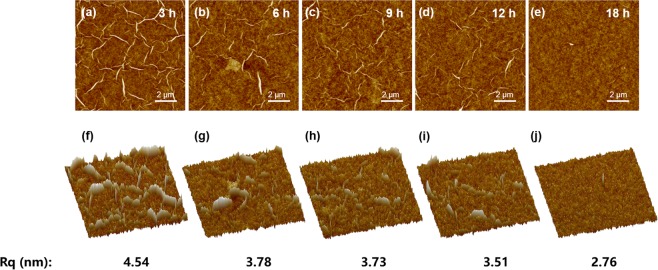
Morphology evolution of rGO films made from different graphene oxide solution under various sonication times. AFM images (**a**–**e**) and corresponding 3D-mode AFM images (**f**–**j**) of rGO films.

The graphene electrode used in this work was made of rGO film prepared by our previous reported method. The evolution of surface morphology in rGO film made from GO precursor under five diverse sonication time is clearly shown in Fig. 1. The atomic force microscope (AFM) images show the top surface of 3 h rGO is covered with dense sharps, resulting in a relative higher surface roughness, while its counterparts (18 h rGO) demonstrates a smoother surface without obvious sharp shape, indicating that the surface roughness of rGO film decreases with the sonication time increase, with a value of 4.54 nm at 3 h, 3.78 nm at 6 h, 3.73 nm at 9 h, 3.51 nm at 12 h, and 2.76 nm at 18 h. The rGO films with the highest and lowest roughness are referred as rough rGO (r-rGO) and smooth rGO (s-rGO), respectively. Therefore, the surface morphology of rGO film could be effectively controlled by adjusting sonication time of GO solution.

**Figure 2:**
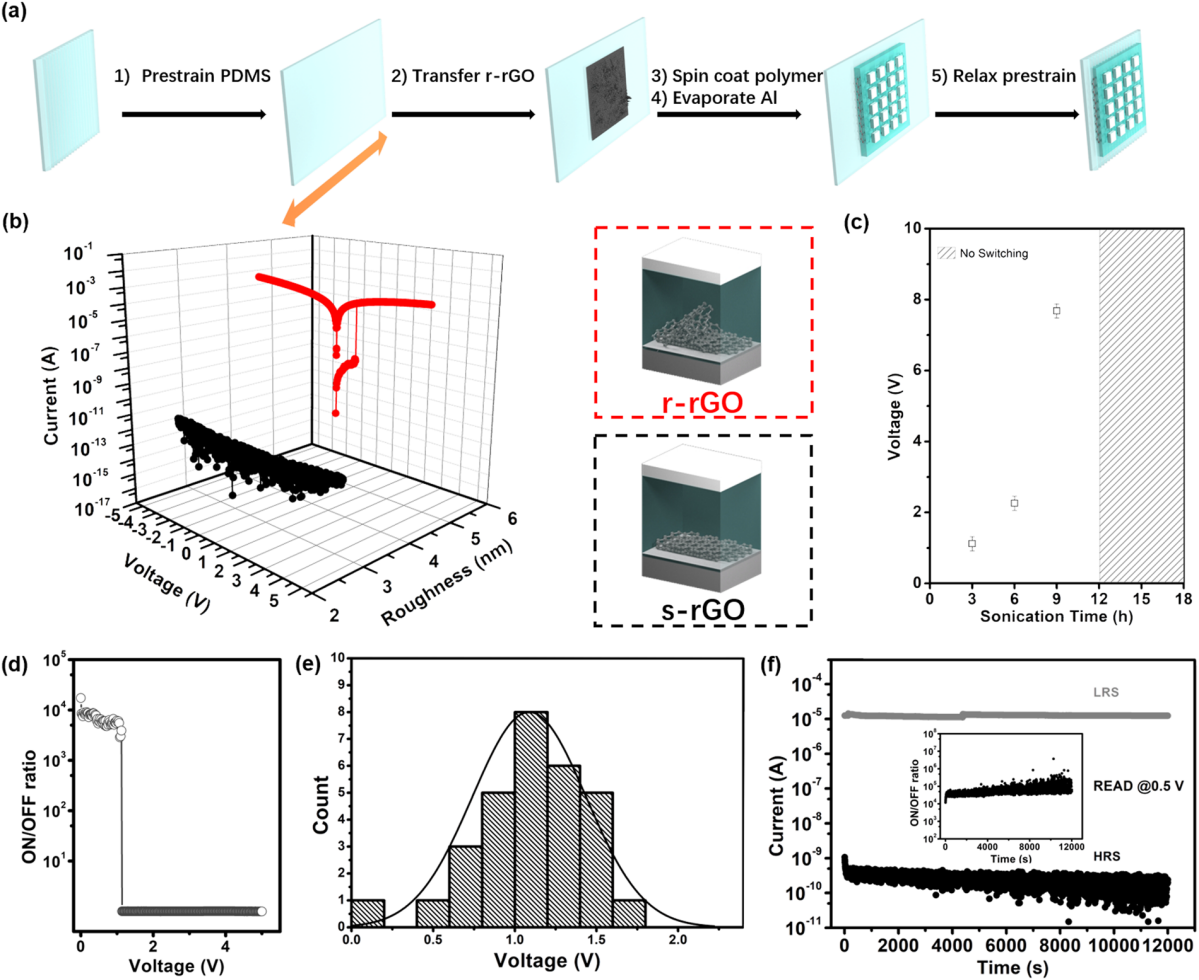
Experimental analysis of the fabricated memory. (**a**) The fabrication process of stretchable polymer memory devices. (**b**) The I–V characteristic of r-rGO/PVK/Al and s-rGO/PVK/Al. (**c**) The sonication time dependence of switching voltage. (**d**) ON/OFF ratio as a function of forward bias. (**e**) Statistics histograms of the switching voltages of r-rGO/PVK/Al memory devices from 30 memory cells. (**f**) The retention ability of r-rGO/PVK/Al memory device at reading voltage of +0.5 V.

The fabrication procedure of stretchable polymer memory devices comprising pure conjugated polymer active layer and r-rGO electrodes is posed in Fig. 2a (see the Experimental Section for details). Briefly, r-rGO film with a sheet resistance of 1000 Ω/sq. was detached and transferred onto target pre-strained PDMS substrate. Then, semiconductive polymer PVK was spin-coated onto r-rGO electrodes, followed by drying in oven. Finally, 200 nm top Al electrodes were thermally evaporated onto polymer layer and stretchable polymer devices were obtained after carefully relaxing the pre-strained system. Similarly, s-rGO was used to replace r-rGO and underwent the identical procedure to fabricate a reference device in order to study the effect of surface morphology of rGO electrode on device performance.

To investigate the memory effects of fabricated polymer devices, the current-voltage (I–V) characteristic of the r-rGO/PVK/Al device was measured and shown in the red dots curve of Fig. 2b. The I–V curves showed a typical electrical bistable phenomenon. The applied voltage was swept with a step of 0.02 V in a cycle from 0 to 5 V and then from 5 to 0 V to realize the electrical transition of device from initial low conductivity state to high conductivity state. Initially, in the range of 0–1.1 V, our device exhibited a high resistance state (HRS, labelled as OFF state). The current increased abruptly at about 1.1 V, indicating a transition from HRS to the low resistance state (LRS, labelled as ON state) of our device. Obviously, once the ON state was achieved, the ON state was maintained even in the absence of power, indicating a non-volatile nature of this memory device. Importantly, the device cannot regain the OFF state during a large negative voltage sweep, indicative of a nonvolatile write-once-read-many times (WORM) characteristic. Moreover, the measured high ON/OFF current ratio of ~10^4^ (Fig. 2d) within individual memory cell, promising low misreading probability in read operation. In contrast, as shown in black dots curve of Fig. 2b, no resistive switching behavior was observed in the reference device with a configuration of s-rGO/PVK/Al. By studying the effect of surface roughness (induced by sonication time) on the memory performance, results showed that the devices performed switching behavior from high resistance state to low resistance state with the ultrasonication time of GO precursor ranging from 3~9 h. Figure 2c demonstrated that the sonication time dependence of switching voltage, indicating that the switching voltage increase with the sonication time increase. However, this WORM switching behavior disappeared as the ultrasonication time applied to the GO precursor overwhelmed 12 h, suggesting that surface sharp morphologies of rGO electrodes play a crucial role in resistive switching behaviors of polymer memory devices, which was experimentally confirmed by works we demonstrated previously.

To evaluate the reproducibility and stability of the polymer memory device, other memory performances, including operation voltage distribution and retention abilities, were measured and investigated systematically. Figure 2e shows that the statistical distribution of switching voltage was calculated from 30 randomly selected memory cells, with an average value of 1.1 V, which is lower than that in most of previous reported PVK memory devices^26–28^, suggestive of their potential use in ultralow power dissipation electronics. Figure 2f shows the retention property of r-rGO/PVK/Al memory, from which a negligible degradation of the electrical conductivity for both ON and OFF state was observed even after considerable duration of 12000 s with a readout voltage of 0.5 V, indicative of the nonvolatile feature and long-time stability of the polymer memory device.

**Figure 3:**
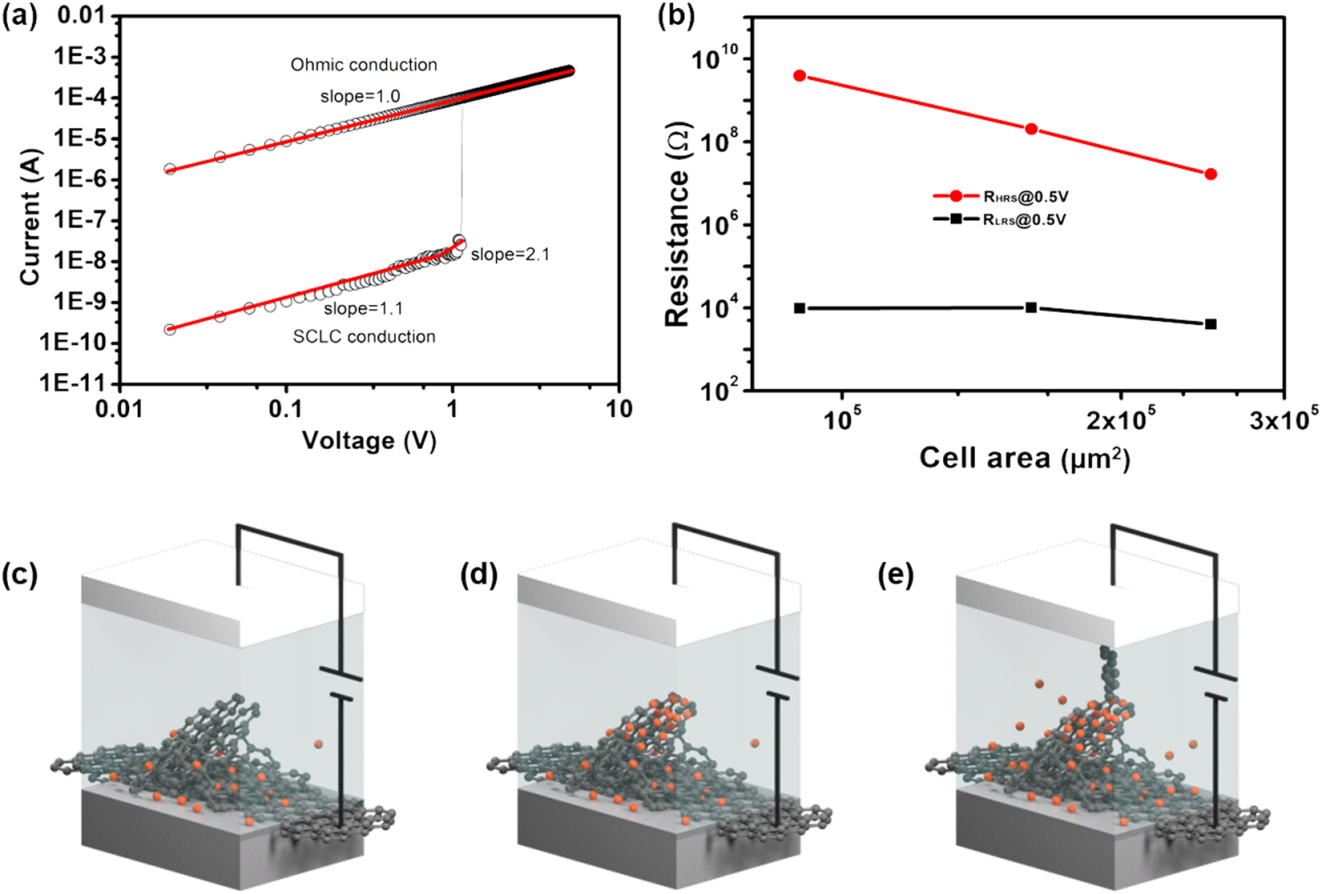
Schematic band diagrams to propose the resistive switching model for memory. (**a**) The plot of current as function of applied voltage for the WORM memory device in the positive sweep. (**b**) The cell area dependence of resistances in HRS and LRS. Schematic diagrams (**c**–**e**) of the proposed resistance switching mechanism for r-rGO/PVK/Al memory device: (**c**) the initial state (OFF state), (**d**) the low current state (OFF state), (**e**) the high current state (ON state).

To explore the resistive switching mechanism in the device, the I–V curves were re-plotted in the log scale (Fig. 3a). In the initial OFF state, from 0 to 0.8 V and from 0.8 to 1.1 V could be fitted well to two straight lines with slope of 1.16 and 2.11, respectively. This phenomenon indicates that the charge transport behavior at relative low voltage follows a denatured trap-controlled space-charge limited conduction (SCLC) (a classical SCLC consists of three portions: an initially Ohmic region (I ∝ V), a trap limited region (I ∝ V^2^), and finally the steep current increase region (I ∝ V^n^)), which is consistent with the previous results^29,30^. Once the voltage exceeded 1.1 V, the device transferred from OFF state into the ON state, Ohmic conduction dominated the charge transportation^31^.

On the basis of the above results, a proposed switching mechanism of the memory device was schematically depicted in Fig. 3c–e. In the low voltage sweep, current was generated by thermally generated free carriers in PVK, which is greater than the injected charge, Ohmic behavior is observed. While the thermally generated free carriers are not sufficient to trigger conductive channels. Thus the bit cell maintains the OFF state. As the voltage increases, part of the injected charges accumulates around the peaks of sharps as others continue to be injected, resulting in a trap limited model^32,33^. When the peaks of sharps accumulated enough carriers, higher internal field was generated to form conductive channels within PVK layer with the resistive switching from OFF state to ON state, which might be attributed to the irreversible formation of carbon-rich filaments in PVK active layer. Accordingly, the device remained in the ON state under a applied reverse voltage. Generally, the sharp surface induced carbon-rich filaments are distinct with conventional metal filaments. The metal filaments normally leads to Flash memory effect since metal filaments are easy to rupture under reverse voltage^29,34^.

In order to further discuss the properties of carbon filaments triggered by sharps, the dependence of device resistance on memory cell area was tested (Fig. 3b). In HRS, the resistance is inversely proportional to the cell area, which is originated from the uniform flow of current through the whole memory cell. In contrast, the resistance in the LRS is independent of the area of the memory cell, indicating that the low resistance level is effectively controlled by local conductive channel, which is composed of carbon conductive filament.

**Figure 4:**
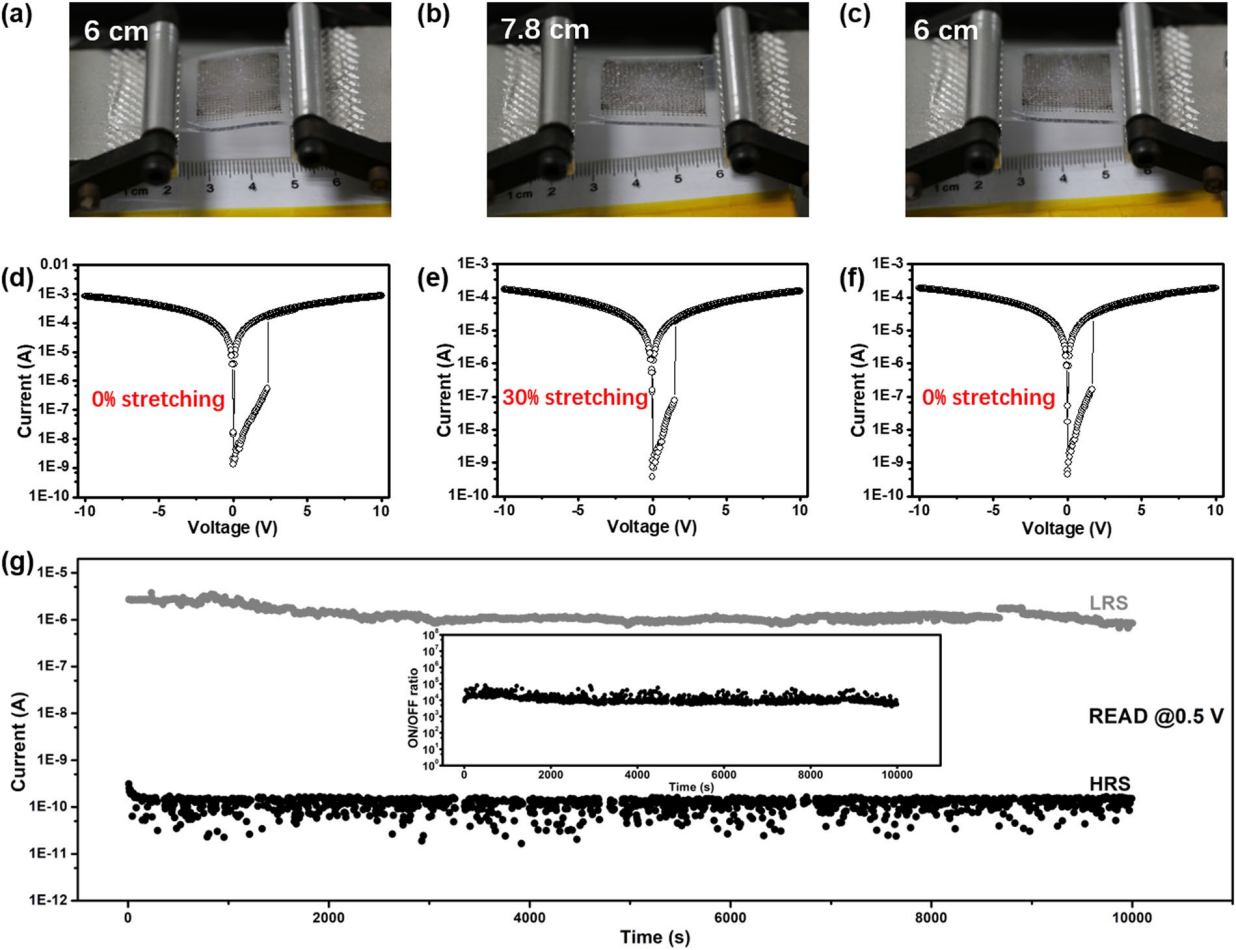
Optical photograph and I–V characteristic curves of stretchable devices in three states. (**a**,**d**) 0% stretching. (**b**,**e**) 30% Stretching. (**c**,**f**) 0% stretching. (**g**) The retention ability of stretchable memory device under the 10% stretching.

Furthermore, in order to assess the stretchability of polymer memory device under tensile force, the electrical characterization of PDMS/r-rGO/PVK/Al memory devices were performed under stretching (Fig. 4d–f). Inset images in Fig. 4a–c are optical photos of the stretchable memory device in 0% stretching, 30% stretching and recovering condition, respectively. An electrical transition from an OFF state to an ON state was also observed, with the current increased abruptly at 2.3 V. After the resistive switching, the memory device maintained at the ON state during the subsequent sweep. The I–V curves of the device undergoing stretching (Fig. 4e) and recovering from stretching (Fig. 4f) were very similar to that of a pristine device (Fig. 4d), indicating high stretchability and electrical stability of our memory device. Figure 4g demonstrated that both resistance state (LRS) and high resistance state (HRS) could maintain after being programmed under the 10% stretching and exhibited negligible fluctuation compared to that before stretching. To further extend the stability test of memory device, a retention measurement was carried out as the device underwent at 318 K during 30000 s with 30% stretching (Fig. S1). Moreover, nonnegligible fluctuations for OFF state was observed as the device underwent 30% stretching. Impressively, there is no obvious degradation of the retention performance in both of the LRS and HRS during the measurement exceeding 10^4^ s.

**Figure 5:**
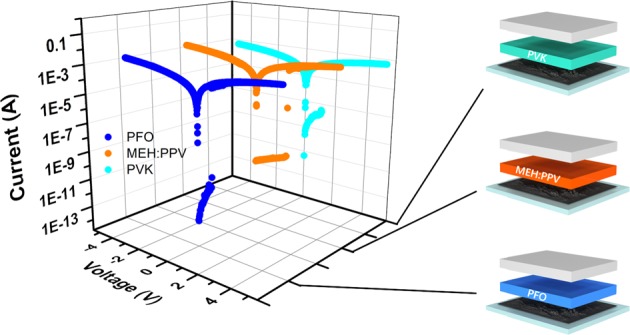
Schematic diagram and I–V characteristics of the r-rGO/PFO/Al, r-rGO/MEH:PPV/Al and r-rGO/PVK/Al.

Finally, to illustrate the generality of our strategy, two other polymer materials were employed as the active layer, such as polymer MEH:PPV and PFO. I–V curves of the two devices were demonstrated in blue dots curve and orange dots curve of Fig. 5 respectively. Impressively, these devices exhibited similar nonvolatile WORM memory characteristic with high ON/OFF ratios and low switching voltages as we expected, confirming the strategy is an effective and universal way to realize high performance stretchable polymer memory device compatibility comprising pure conjugated polymers and r-rGO conductive electrodes.

## Discussion

In summary, a universal strategy for high performance nonvolatile stretchable pure polymer memory devices with a architecture of r-rGO/semiconductor polymers (PVK, MEH:PPV, and PFO)/Al has been demonstrated. The r-rGO/PVK/Al device exhibits nonvolatile WORM-type memory, with low switching voltage of 1.1 V, high ON/OFF ratio of 10^4^ and desirable long retention time over 12000 s. The switching behavior of the device is ascribed to carbon-rich filaments induced by sharp rGO surface. Moreover, this stretchable data storage system has outstanding mechanical properties with a retractable 30% elongation. Furthermore, this facile fabrication process enables excellent reproducibility. This strategy combining such advances in stretchable polymer memory devices with simple structure, common materials, superior reproducibility, outstanding performance, and reliable stretchabiblity pose great potential in permanent storage applications toward stretchable electronics.

## Methods

### Material preparations

In order to prepare the memory cell active material, PVK (300 mg) was dissolved into chlorobenzene (10 ml) to make the solution. For preparation of the sharp rGO surface, The GO dispersion in methanol (0.5 mg/mL) was spin-coated on the pre-cleaned Si/SiO_2_ substrate (SZJXTech, China) at 2500 rpm. GO film followed by annealing at 1000 °C under an Ar_2_/H_2_ mixture for 2 h to obtain the rGO film. Importantly, the r-rGO and s-rGO can be fabricated by tailoring ultrasonic time of GO suspensions^31,35^.

### Fabrication of stretchable polymer memory devices

R-rGO film was fabricated on Si/SiO_2_ substrate. Firstly, the PMMA suspension was spin-coated onto rGO film at 3000 rpm, followed byannealling in air at 170 °C for 30 min. Then the film was etched in NaOH, the film was transferred to a pre-strained PDMS substrate on a hot plate with temperature of 60 °C for 180 min. The stretchable PDMS/r-rGO electrode can be achieved by purged PMMA with acetone solution. Polymer memory devices were fabricated on the prepared PDMS/r-rGO electrode. The active layer is semiconductor polymers, including PVK, MEH:PPV and PFO. The polymer suspension (PVK in chlorobenzene, MEH:PPV in chlorobenzene, PFO in chlorobenzene, 10 mg/mL) was spin-coated on rGO film at 1000 rpm, followed by annealling in air at 100 °C for 30 min. Finally, the 200 nm top Al electrode (0.2 × 0.2 mm^2^) was thermally evaporated. Importantly, the thickness of polymer active layer can be modulated by tailoring the concentration of polymer suspension or speed of spin coating. Finally, the stretchable polymer memory devices were fabricated by relaxing the pre-strained structure.

### Characterization

The atomic force microscopy (AFM) images were obtained by using a Dimension 3100 (veeco, CA) in tapping mode under ambient conditions. The I–V characteristic of the device were measured by a keithley 4200 semiconductor parameter analyzer under ambient conditions. The sheet resistance was measured by a four-probe instrument.

## Supplementary information

A Universal Strategy for Stretchable Polymer Nonvolatile Memory via Tailoring Nanostructured Surfaces
