# Supplementary material for: A Universal Strategy for Stretchable Polymer Nonvolatile Memory via Tailoring Nanostructured Surfaces
Source: Sci Rep. 2019 Jul 17;9:10337. doi: 10.1038/s41598-019-46884-4 (PMC6637107; doi:10.1038/s41598-019-46884-4)
Supplement: Supplementary file 1 — A Universal Strategy for Stretchable Polymer Nonvolatile Memory via Tailoring Nanostructured Surfaces [file 41598_2019_46884_MOESM1_ESM.pdf]

# A Universal Strategy for Stretchable Polymer Nonvolatile Memory via Tailoring Nanostructured Surfaces

Chaoyi Ban[1], Xiangjing Wang[1], Zhe Zhou[1], Huiwu Mao[1], Shuai Cheng[1], Zepu Zhang[1], Zhengdong Liu[1], Hai Li[1], Juqing Liu[*1], Wei Huang[*1, 2, 3]

[1]Key Laboratory of Flexible Electronics (KLOFE) & Institute of Advanced Materials (IAM), Nanjing Tech University (NanjingTech), 30 South Puzhu Road, Nanjing 211816, China. [2]Shaanxi Institute of Flexible Electronics (SIFE), Northwestern Polytechnical University (NPU), 127 West Youyi Road, Xi'an 710072, China. [3]Key Laboratory for Organic Electronics and Information Displays & Institute of Advanced Materials (IAM), SICAM, Nanjing University of Posts & Telecommunications, 9 Wenyuan Road, Nanjing 210023, China. Correspondence and requests for materials should be addressed to J.Q.L (email: iamjqliu@njtech.edu.cn) or to W.H. (email: wei-huang@njtech.edu.cn)

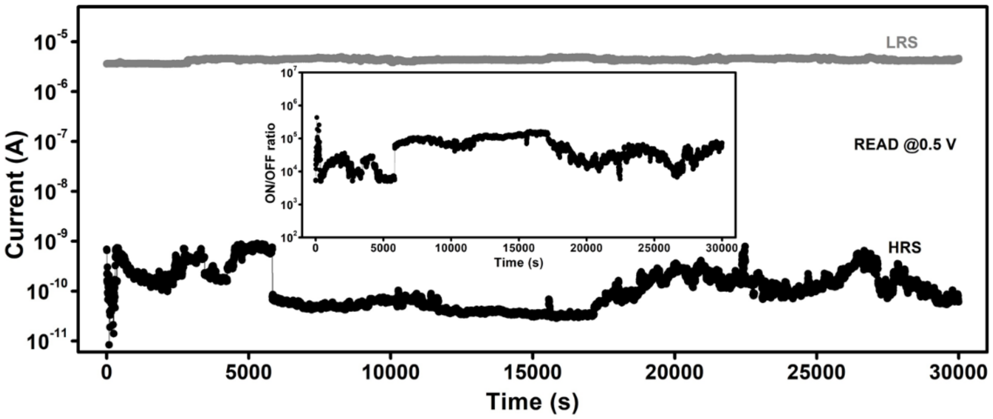


**Figure 1 |** The retention ability of stretchable memory device under the 30% stretching.
